# γ Peptide Nucleic Acid-Based miR-122 Inhibition Rescues Vascular Endothelial Dysfunction in Mice Fed a High-Fat Diet

**DOI:** 10.1021/acs.jmedchem.1c01831

**Published:** 2022-02-08

**Authors:** Ravinder Reddy Gaddam, Karishma Dhuri, Young-Rae Kim, Julia S. Jacobs, Vikas Kumar, Qiuxia Li, Kaikobad Irani, Raman Bahal, Ajit Vikram

**Affiliations:** † Department of Internal Medicine, Carver College of Medicine University of Iowa, Iowa City, Iowa 52242, United States; ‡ Department of Pharmaceutical Sciences, University of Connecticut, Storrs, Connecticut 06269, United States

## Abstract

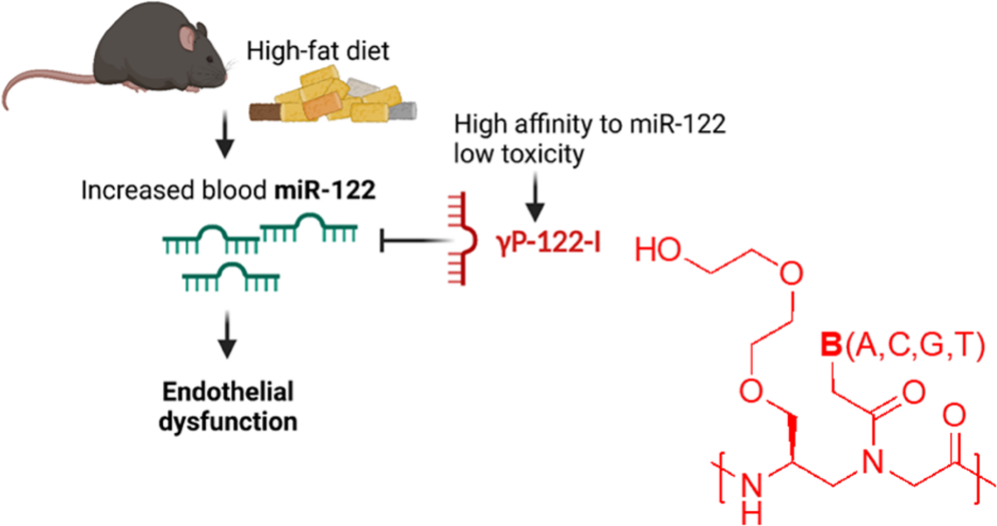

The blood levels of microRNA-122 (miR-122) is associated with the severity of cardiovascular disorders, and targeting it with efficient and safer miR inhibitors could be a promising approach. Here, we report the generation of a γ-peptide nucleic acid (γPNA)-based miR-122 inhibitor (γP-122-I) that rescues vascular endothelial dysfunction in mice fed a high-fat diet. We synthesized diethylene glycol-containing γP-122-I and found that its systemic administration counteracted high-fat diet (HFD)-feeding-associated increase in blood and aortic miR-122 levels, impaired endothelial function, and reduced glycemic control. A comprehensive safety analysis established that γP-122-I affects neither the complete blood count nor biochemical tests of liver and kidney functions during acute exposure. In addition, long-term exposure to γP-122-I did not change the overall adiposity, or histology of the kidney, liver, and heart. Thus, γP-122-I rescues endothelial dysfunction without any evidence of toxicity *in vivo* and demonstrates the suitability of γPNA technology in generating efficient and safer miR inhibitors.

## Introduction

Hypertension is a common impediment in patients with type 2 diabetes, and both diabetes and hypertension are individually associated with increased risk of cardiovascular events.^1^ Patients with diabetes are at higher risk of nondipping hypertension^2−4^ and heart/kidney failure.^5−12^ Current treatment approaches fail to decrease unwanted cardiovascular outcomes in these patients.^13,14^ In patients with diabetes, the risk of hypertension is preceded and predicted by endothelial dysfunction.^15^ One promising approach to effectively combat endothelial dysfunction involves targeting microRNAs (miRs).^16^ Specifically, miR-122-5p (miR-122) is considered a target because of its increased levels in patients with diabetes and/or obesity,^17−23^ which correlates with severity of cardiovascular disorders.^24−27^ miR-122 is primarily expressed in the liver and released into the blood.^28−30^ Its release into the blood is increased in the contexts of obesity, non alcoholic fatty liver disease, and liver toxicity.^31,32^ We recently demonstrated that in endothelial cells, miR-122 regulates expression of the proinflammatory miR-204, a molecule that promotes vascular endothelial dysfunction.^33^ Also, a recent report established that the inhibition of miR-122 prevents atherosclerosis in ApoE^-/-^ mice, which are hypercholesterolemic and spontaneously develop atherosclerosis.^34^ Therefore, we postulate that the systemic inhibition of miR-122 will prevent the development of endothelial dysfunction.

miR inhibitors are DNA analogues that consist of either a natural negatively charged phosphodiester backbone (conventional) or a modified phosphodiester backbone.^35^ The negatively charged backbones of inhibitors interact nonspecifically with proteins, prolonging their half-lives and leading to adverse outcomes because of nonspecific accumulation in the tissues.^36−38^ The inhibitors with chemically modified phosphodiester backbone are superior, demonstrating robust enzymatic stability and higher binding affinity.^39^ Among these, peptide nucleic acids (PNAs) have gained substantial attention as potential miR inhibitors in recent years.^40^ PNAs are synthetic DNA mimics in which the phosphodiester backbone is replaced with a *N*-(2-aminoethyl) glycine backbone,^41^ are enzymatically stable, and have a high binding affinity for target sites.^42^ Although the charge neutrality of PNAs has the benefit of reducing their nonspecific interactions with serum proteins, these early (classical) forms have the disadvantages of being poorly soluble in water. Because of this limitation, the classical PNAs did not progress as the molecules of choice.^38,39,43−45^ The next-generation PNAs that include modification at the γ-position of the nucleobase, known as γPNAs, form preorganized helical structures by engaging γ position of the backbone as the stereogenic center.^46^ This preorganization confers even stronger binding affinity for the target RNA than that of the classical PNAs.^47^ A second improvement in γPNAs is that they contain diethylene glycol units, which increase their solubility and hence their biocompatibility.^48^ In prior studies, we established that γPNAs have improved water solubility and increased binding affinity for target RNA sites. In addition, γPNAs neither aggregate nor adhere to proteins nonspecifically.^49,50^ Collectively, the features of the γPNA—a charge-neutral backbone, high water solubility, and a high binding affinity for miRs—make them excellent candidates for gene targeting and editing-based applications.^49,50^ γPNAs have been established as effective tools in several biological and biomedical applications: genetic barcoding,^51^ nanotechnology-mediated delivery,^52^ gene editing,^53−56^ and gene targeting.^57,58^ However, the γPNA technology has not been tested for generating miR inhibitors that inhibit cardiovascular disorders.

Here, we tested the effectiveness of the γPNA technology in inhibiting miR-122 activity and rescuing endothelial dysfunction in prediabetic mice. Our results demonstrate that a γPNA-based miR-122 inhibitor efficiently inhibits miR-122, improves glycemic control and endothelial dysfunction in prediabetic mice, and is safe in short- and long-term use.

## Results

### Design and Characterization of γP-122-I

**Figure 1:**
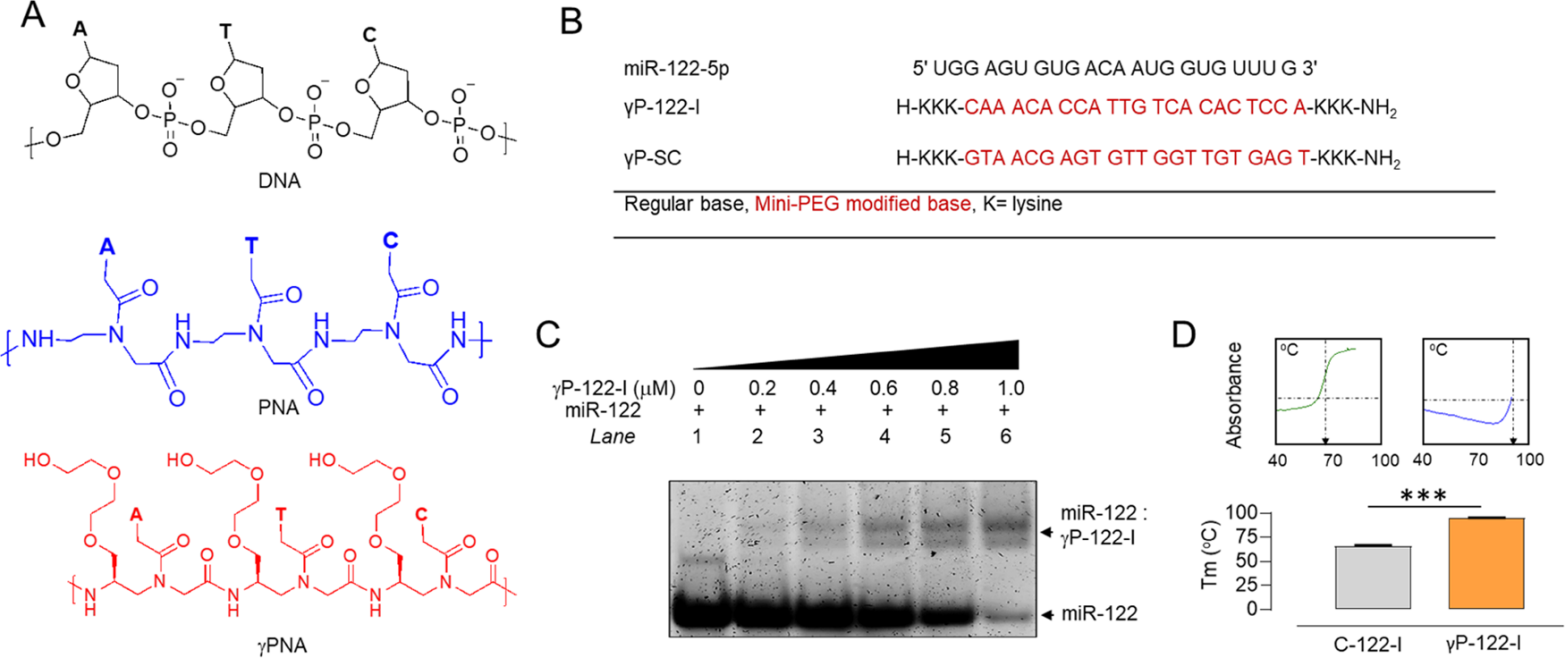
Binding of γP-122-I to miR-122. (A) Chemical structures of DNA, PNA, and γPNA oligomers containing nucleobases (A, T, and C). In γPNA, the diethylene glycol group is included at the γ position. (B) Sequences of miR-122-5p, γP-122-I, and γP-SC. All nucleobases in γP-122-I and γP-SC were γ modified. (C) Gel-shift assay assessing miR-122 (1 μM) binding by γP-122-I over a range of concentrations. Bands were visualized using SyBr gold stain. (D) Melting temperatures (*T*_m_) of heteroduplexes of miR-122 with C-122-I and γP-122-I. The panels above show the typical melting curves for each. *n* = 3. ***p < 0.001 vs C-122-I. Data are shown as mean, and error bars represent the standard error of the mean (SEM). C-122-I; Commercially available miR-122 inhibitor.

To test the effectiveness in targeting miR-122, we designed and synthesized both miR-122-targeting and scrambled control γPNA oligomers. The γ-modified nucleobases contained diethylene glycol at the γ position. To improve the solubility of PNA and its binding to miR-122, we appended lysine to both the 5′ and 3′ ends of γPNA, based on our prior study showing that lysine increases the binding affinity of PNAs.^33^ We synthesized diethylene glycol-containing γPNA-based miR-122 inhibitors (Figure 1A,B, γP-122-I) and scrambled controls (Figure 1B, γP-SC). γPNAs were synthesized using established solid-phase protocols,^59^ and their quality was determined by high-performance liquid chromatography (HPLC) and matrix-assisted laser desorption/ionization (MALDI) spectrometry (Figure S1). We next determined the binding of γP-122-I with miR-122 by gel-shift assay and found that the amount of miR-122 bound by γP-122-I was dependent on the concentration of the latter (Figure 1C). The binding affinity of γP-122-I for miR-122 was analyzed by thermal denaturation of heteroduplexes formed between the inhibitor and miR-122. For comparison, we also evaluated the denaturation of heteroduplexes formed from a commercially available miR-122 inhibitor (C-122-I) and the same target construct. We found that the temperature at which γP-122-I:miR-122 heteroduplexes were denatured was significantly higher (*T*_m_ = 95 ± 0.2 °C) than that at which this occurred for C-122-I:miR-122 heteroduplexes (*T*_m_ = 66 ± 0.8 °C) (Figure 1D).

**Figure 2:**
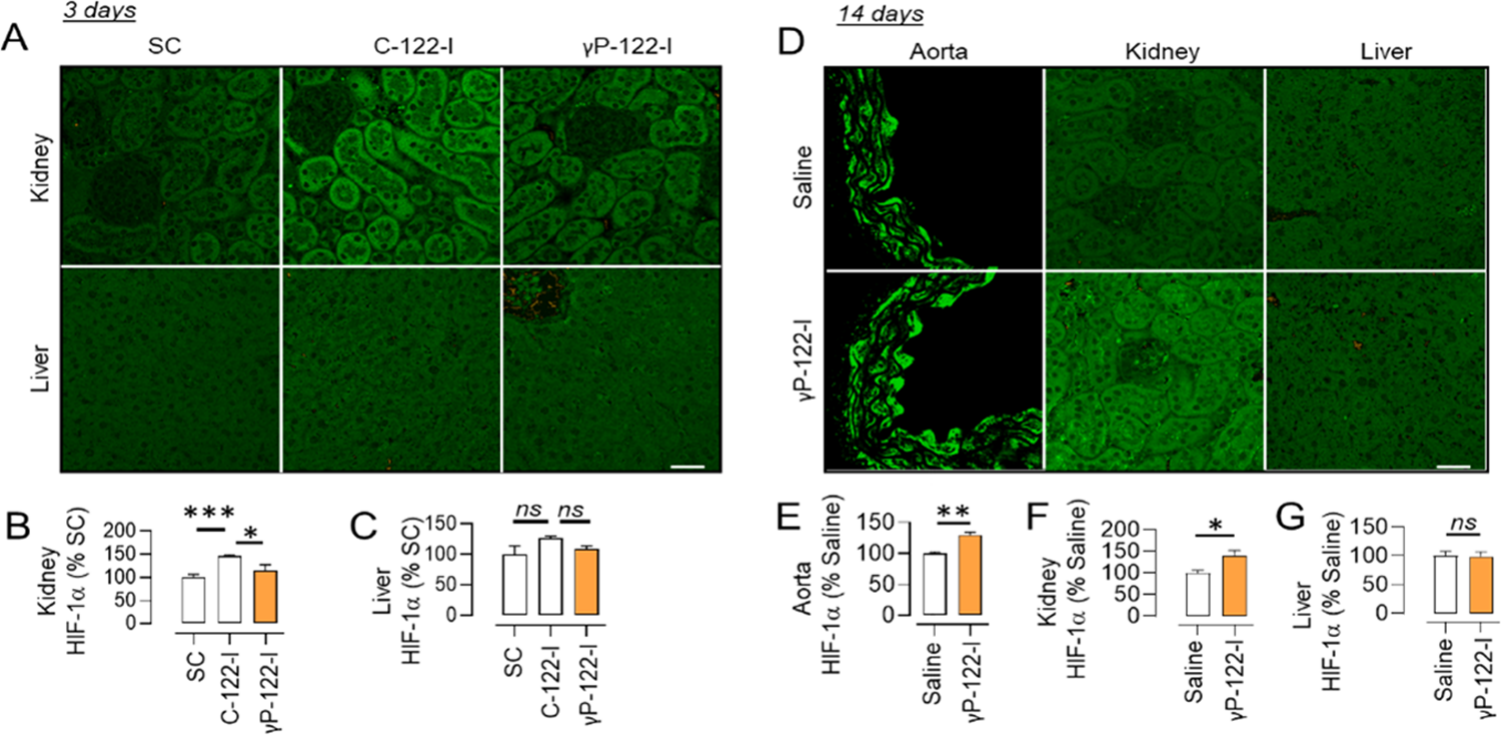
Effects of γP-122-I on the expression of HIF-1α. (A–C) Effects of 3 day application of a C-122-I and γP-122-I at 62.5 nmol kg^–1^ (0.25 and 0.5 mg kg^–1^, respectively) on HIF-1α expression in the kidney (A,B) and liver (A,C). *n* = 3–4, ×20 fields. (D) Effects of 14 day administration of γP-122-I (1.25 μmol kg^–1^) on HIF-1α expression in the aorta, kidney, and liver. (E–G) Quantification of HIF-1α in aorta (E), kidney (F), and liver (G). *n* = 3–9, ×20 fields. *p < 0.05, **p < 0.01, and ***p < 0.001 vs indicated group. The scale bar represents 20 μm. Data shown as mean and error bars represent SEM.

Our rationale for developing γP-122-I is based on its anticipated biocompatibility, which depends on its lower nonspecific tissue retention. We thus evaluated the effects of this inhibitor on the hepatic and renal expression of the miR-122 target HIF-1α (a surrogate marker of miR-122 inhibition).^60,61^ Male mice were injected with either γP-122-I or C-122-I (62.5 nmol kg^–1^) for 3 days, and then HIF-1α expression was assessed. We found that γP-122-I had less effect than C-122-I on HIF-1α expression in the kidney (Figure 2A,B). At this dose, we did not observe an increase in HIF-1α expression in the liver with either γP-122-I or C-122-I (Figure 2A,C). As the liver expresses 100–1000-fold more miR-122 than serum, the vasculature, and the kidney,^28^ we reasoned that this dose and the duration of treatment (γP-122-I or C-122-I) were inadequate to change HIF-1α. Thus, we tested γP-122-I at a higher dose (1.25 μmol kg^–1^) and for a longer duration (14 days). This led to an increase in HIF-1α in aorta and kidney but still failed to induce a significant change in the liver (Figure 2D–G). The biodistribution of γP-122-I was determined using TAMRA-tagged γP-122-I. We found that following intraperitoneal injection, its concentration peaks in serum and urine at 0.5 and 2 h, respectively (Figure S2).

### γP-122-I Rescues Endothelial Dysfunction in Mice Fed an HFD

**Figure 3:**
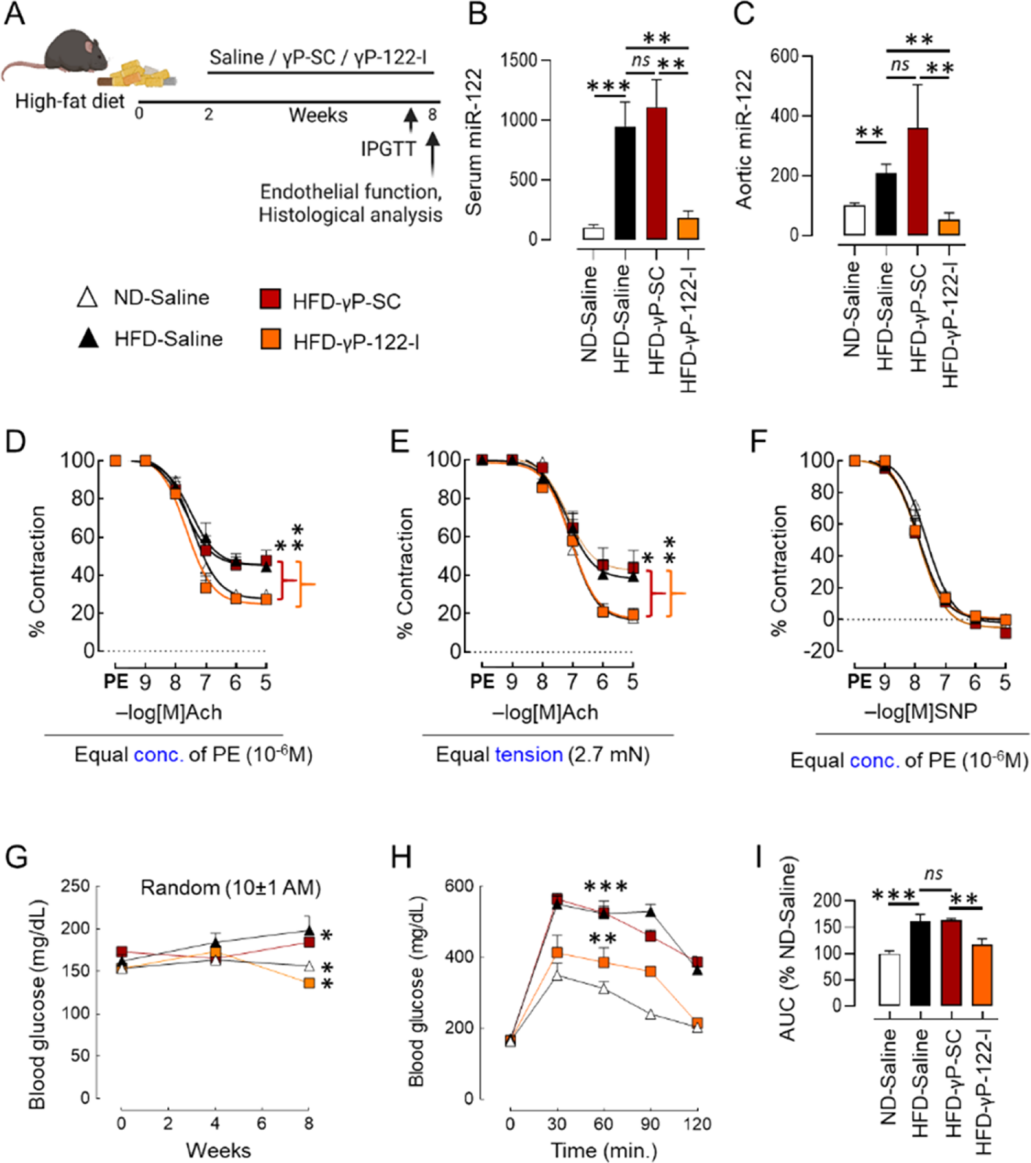
Effects of systemic administration of γP-122-I on HFD-triggered defects in glucose tolerance and endothelial function. (A) Schematic showing the timing of HFD feeding, treatment with γP-SC and γP-122-I, and termination of the experiment. It was created with BioRender.com. (B,C) Effects of γP-122-I (0.25 μmol kg^–1^ or 5 mg kg^–1^) on HFD-triggered upregulation of miR-122 in the serum (B, *n* = 4–9) and aorta (C, *n* = 3–6). (D) Effects of γP-122-I on HFD-triggered impairment of acetylcholine-induced vasorelaxation of aortic rings that had been precontracted (treatment with phenylephrine: 10^–6^ M). *n*(*N*) = 6(2)–18(6). (E) Effects of γP-122-I on HFD-triggered impairment of acetylcholine-induced vasorelaxation of aortic rings that had been precontracted to equal tension (2.7 mN; treatment with phenylephrine). *n*(*N*) = 8(2)–14(6). (F) Effects of γP-122-I on sodium-nitroprusside (SNP)-induced vasorelaxation of precontracted (treatment with phenylephrine: 10^–6^ M) aortic rings. *n*(*N*) = 8(2)–24(6). The replicate for (D)–(F) is shown as *n*(*N*), where *n* is the aortic ring number and *N* is the mice number. (G,H) Effects of treatment with γP-122-I on the random blood glucose level (G) and glucose disposal during intraperitoneal glucose tolerance tests (H). *n* = 4–8. (I) Quantitation of the area under the curve (AUC) in (H). *n* = 5–10. Regression analysis data for *XY* plots were used to determine the significance of the difference. *p < 0.05, **p < 0.01, and ***p < 0.001 vs indicated group. Data are shown as mean, and error bars represent SEM. PE, phenylephrine; Ach, acetylcholine; and mN, millinewtons.

**Figure 4:**
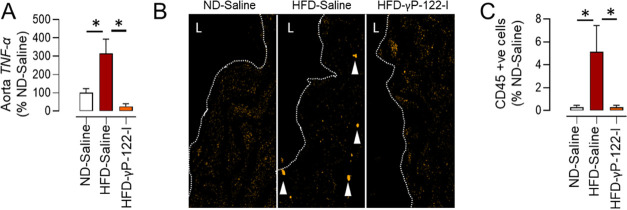
Effects of γP-122-I on HFD-triggered vascular inflammation. (A) As assessed by quantitative polymerase chain reaction (qPCR), the effect of γP-122-I on HFD-triggered upregulation of aortic TNF-α expression. *n* = 4. (B,C) Effect of γP-122-I on HFD-triggered infiltration of CD45-positive cells in the aortic wall (B) and its quantification (C). (magnification ×40). L, lumen. *n* = 7. *p < 0.05 vs indicated group. Data are shown as mean, and the error bar represents SEM.

A calorie-rich diet leads to impaired glycemic control that resembles the early stages of diabetes.^62,63^ Similarly, mice kept on an HFD for 8 weeks develop significant impairment of endothelium-dependent (acetylcholine-mediated) vascular relaxation and glycemic control.^33,64^ Here, we used this prediabetic mouse model to investigate the effects of γP-122-I on endothelial function. We treated HFD-fed mice with γP-122-I at 0.25 μmol kg^–1^ (5 mg kg^–1^) for 6 weeks beginning 2 weeks after the dietary intervention (Figure 3A). Mice fed a normal diet (ND) and HFD-fed mice that received saline or γP-SC served as controls. Feeding of the HFD led to a significant increase in serum and vascular levels of miR-122, and this effect was significantly inhibited in HFD-fed mice that received γP-122-I (Figure 3B,C). We assessed endothelium-dependent (acetylcholine-mediated) vascular relaxation of aortic rings precontracted by treatment with phenylephrine (PE, 10^–6^ M). Aortic rings isolated from HFD-fed mice receiving saline or γP-SC (positive controls) had impaired endothelial function relative to those from mice on the ND and receiving saline (negative controls). The HFD-fed mice receiving γP-122-I displayed significant recovery of endothelial dysfunction (Figure 3D). As diabetic conditions *per se* affect the contractility of blood vessels, we additionally measured the effects of γP-122-I on the acetylcholine-dependent vascular relaxation of aortic rings that were contracted to equal tension (2.7 ± 0.15 mN). We found that in the aorta isolated from the mice receiving γP-122-I, the relaxation was significantly improved (Figure 3E). Sodium nitroprusside (SNP) is a nitric oxide donor and induces endothelium-independent vasorelaxation. In contrast to acetylcholine-induced vasorelaxation, SNP-induced vasorelaxation did not differ in HFD-fed mice receiving γP-SC or γP-122-I, suggesting that γP-122-I improves endothelial function (Figure 3F). Next, we ascertained the effect of γP-122-I on glycemic control and found that it significantly improved random serum glucose levels and blood glucose disposal during the intraperitoneal glucose tolerance test (IPGTT) (Figure 3G–I). We also noticed a significant reduction in body weight but no change in the overall adiposity in HFD-fed mice receiving γP-122-I (Figure S3A,B). In diabetes and obesity, PPAR-α is a target for controlling glycemia and metabolic dysregulation,^65,66^ and miR-122 can regulate PPAR-α.^67^ Therefore, we measured the effect of γP-122-I on hepatic PPAR-α and found that it decreases the HFD-induced PPAR-α upregulation in the HFD-fed mice (Figure S3C). The endothelial dysfunction in HFD-fed mice is associated with vascular inflammation. Therefore, we assessed the effect of γP-122-I on vascular inflammation and found that it reduced an HFD-triggered increase in the aortic expression of *TNF*-α (Figure 4A). CD45 is a marker of hematopoietic cells, and the increased frequency of CD45-positive cells suggests that at least one inflammatory cell type was activated.^68^ Staining of aortic sections for CD45 revealed a significant reduction in the frequency of CD45-positive cells in the aortic wall of γP-122-I-treated versus saline-treated HFD-fed mice (Figure 4B,C). The inflammation could also be mitigated by improving glycemic control and a decrease in body weight, contributing to endothelial dysfunction rescue. To determine the endothelial contribution to the endothelial function of HFD-fed mice treated with the miR-122 inhibitor, we assessed the effects of miR-122 inhibition on eNOS and ERK1/2 in vitro under hyperglycemic conditions. The hyperglycemic conditions decrease eNOS^69,70^ and increase ERK1/2 activation.^71,72^ We found that hyperglycemia (25 mmol L^–1^, 24 h) increases eNOS expression but decreases its activation (p-eNOS) in human umbilical vein endothelial cells (HUVECs), the effect that was partially reversed by the miR-122 inhibition (Figure S4). No significant difference in either expression or activation of ERK1/2 in HUVECS under hyperglycemic conditions was observed, and neither was it affected by the miR-122 inhibition (Figure S4).

### *In Vivo* Toxicity of γP-122-I

**Figure 5:**
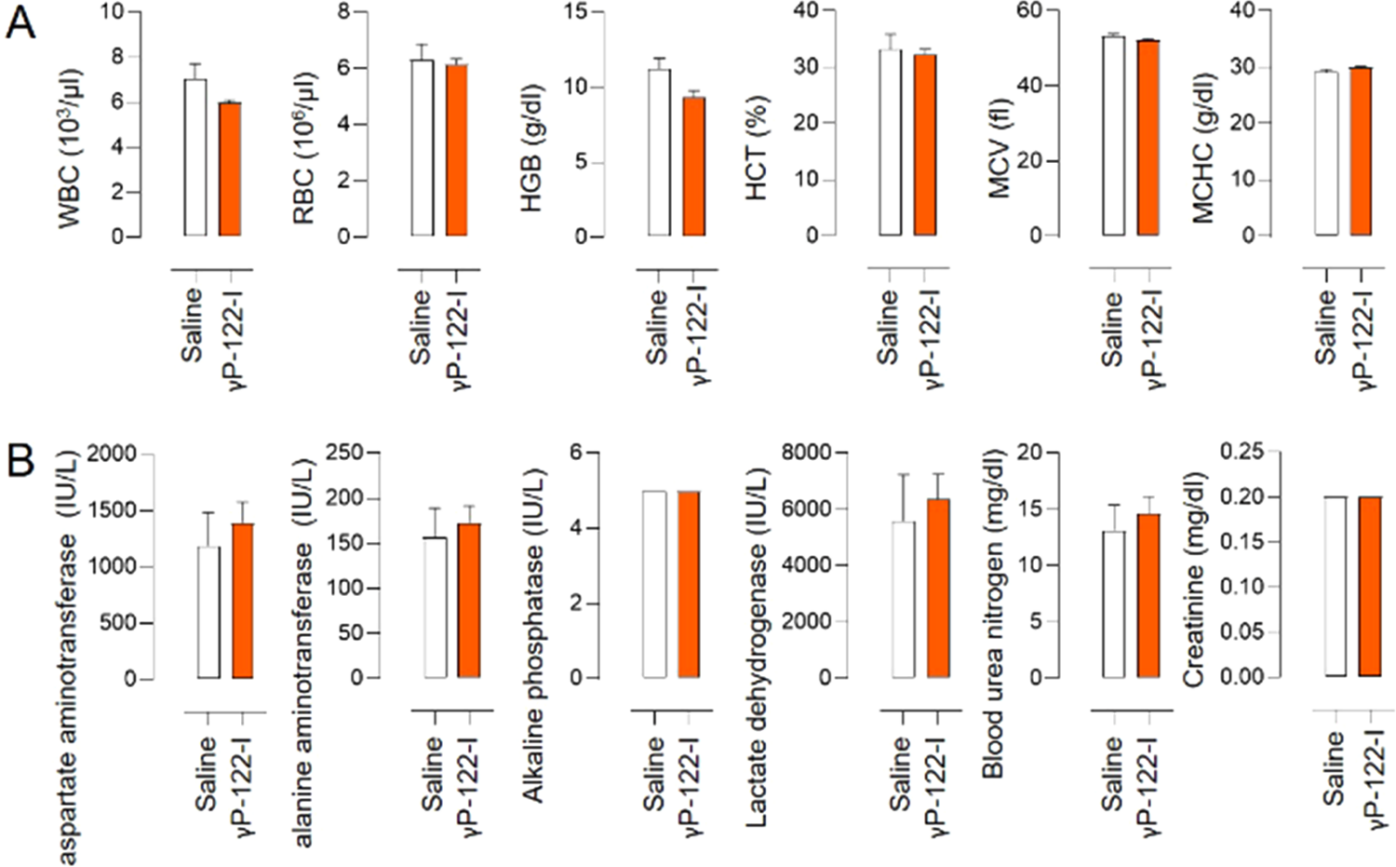
Acute effects of γP-122-I on complete blood counts and on liver and kidney enzyme levels in the blood. (A) White blood cells (WBC), red blood cells (RBC), hemoglobin (HGB), hematocrit (HCT), mean corpuscular volume (MCV), and mean corpuscular hemoglobin concentration (MCHC) levels in the blood of mice that had received either saline or γP-122-I, at 24 h (0.25 μmol kg^–1^) postadministration. *n* = 6. (B) Levels of aspartate aminotransferase (AST), alanine aminotransferase (ALT), alkaline phosphatase (AP), lactate dehydrogenase (LDH), blood urea nitrogen (BUN), and creatinine in blood from mice that had received either saline or γP-122-I at 24 h (0.25 μmol kg^–1^) postadministration. *n* = 6. Data are shown as mean and the error bar represents SEM.

To determine the *in vivo* safety of γP-122-I, we measured its acute (24 h) effects, at the dose of 0.25 μmol kg^–1^, on the complete blood count (CBC) and assessed the blood levels of biochemical indicators of liver and kidney functions. Acute exposure to γP-122-I was not associated with an appreciable difference relative to the control, in terms of white blood cell (WBC) counts, red blood cell (RBC) counts, hemoglobin (HGB) levels, hematocrit (HCT), mean corpuscular volume (MCV), and mean corpuscular hemoglobin concentration (MCHC) (Figure 5A). In addition, the acute exposure of γP-122-I was not associated with significant differences relative to the saline-treated group in terms of liver and kidney functions, as indicated by the levels of aspartate aminotransferase, alanine aminotransferase, alkaline phosphatase, lactate dehydrogenase, blood urea nitrogen, and creatinine (Figure 5B).

**Figure 6:**
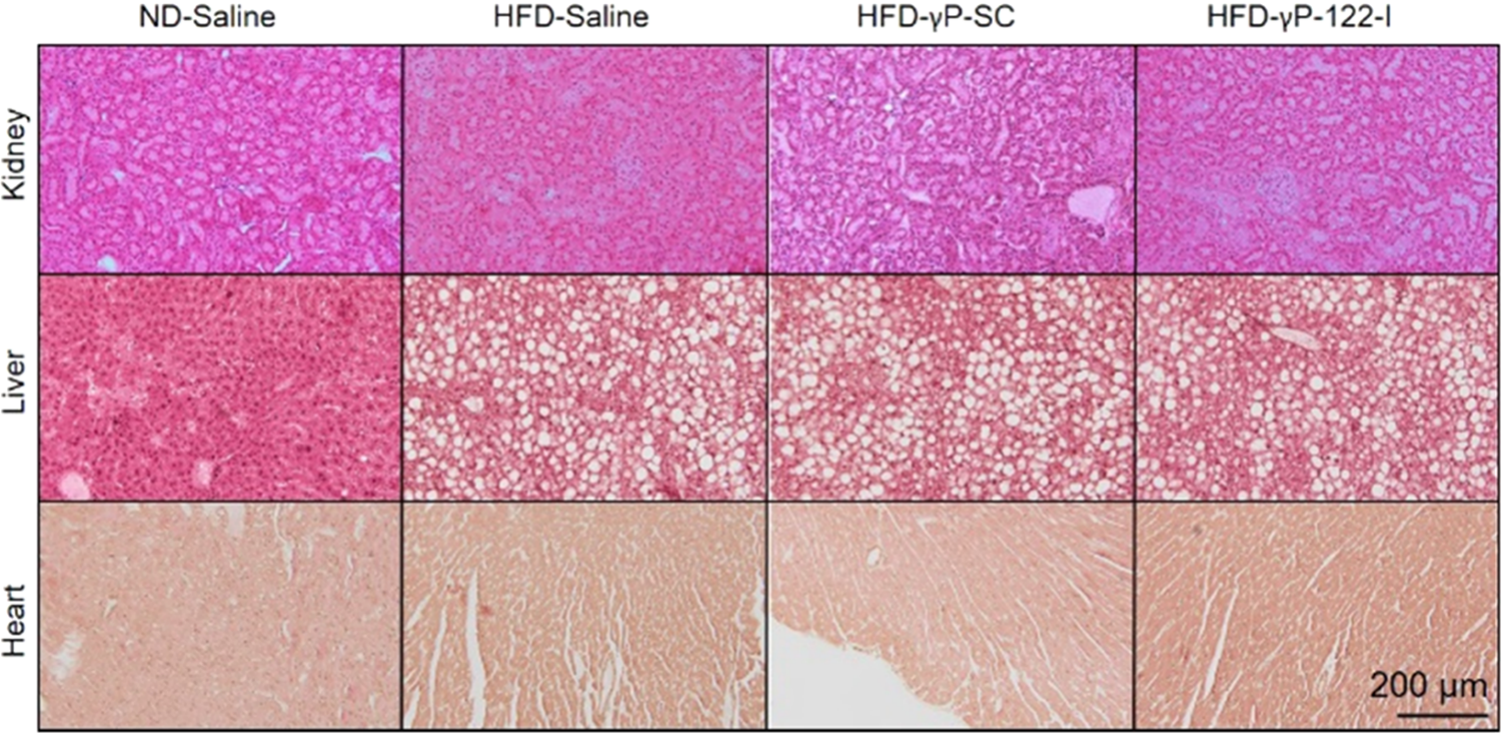
Long-term effects of γP-122-I on tissue histology. Photomicrographs showing hematoxylin and eosin-stained histological sections of kidneys, livers, and hearts of ND-fed mice receiving saline, HFD-fed mice receiving saline, HFD-fed mice receiving γP-SC, and HFD-fed mice receiving γP-122-I. The mice fed an HFD for 8 weeks and were treated with γP-122-I or γP-SC for 6 weeks (0.25 μmol kg^–1^) beginning 2 weeks after the dietary intervention.

Next, we determined the long-term (6 weeks) effects of γP-122-I at the dose of 0.25 μmol kg^–1^ day^–1^ on body weight gain, adiposity, and organ histology (liver, kidney, and heart). We found that the HFD significantly increased body weight and was not affected by the administration of γP-SC for 6 weeks. However, HFD-fed mice that received γP-122-I were slightly leaner than HFD-fed mice that received γP-SC or saline (Figure S3A). The HFD feeding increased adiposity, which was not affected by either γP-SC or γP-122-I (Figure S3B). We compared hematoxylin and eosin-stained histological sections of kidney, liver, and heart from ND-fed mice receiving saline, HFD-fed mice receiving saline, HFD-fed mice receiving γP-SC, and HFD-fed mice receiving γP-122-I. In the cases of kidney and heart, no histological differences were detected across these experimental groups (Figure 6). The liver of HFD-fed mice had a high frequency of vacuolation, a sign of fat deposition. However, the liver of HFD-fed mice treated with γP-122-I was histologically not different compared to that of HFD-fed mice treated with either saline or γP-SC (Figure 6).

## Discussion

In prior studies, inhibiting miR-122 in the context of the hepatitis C virus showed promise.^73−78^ However, the miR-122 inhibitor (RG-101) developed by Regulus Technologies was put on clinical hold due to the development of jaundice.^76,79^ Further, studies in hepatocellular carcinoma patients and mice lacking miR-122 raised concern over the long-term effects of inhibiting miR-122 on hepatic function.^80−82^ In general, the moderate efficacy of previous miR-targeted inhibitors and the associated adverse effects are the critical roadblocks in developing miR therapeutics. Other concerns associated with using miR inhibitors for clinical applications are their moderate efficacy and adverse effects. The latter include prolonging the activated partial thromboplastin time (aPTT; time it takes for clotting to occur), activating the complement cascade, and nonspecific accumulation in tissues. The aPTT depends on the plasma concentration of oligonucleotides and is not clinically significant, as its impact can be weakened by optimizing the delivery regimen.^83−85^ The time of complement cascade activation cannot be predicted based on the properties of an miR inhibitor and must be determined for each. However, the accumulation of nucleic acid analogues, which depends on their negative charges, prolongs the half-life (2–4 weeks) and contributes to adverse outcomes.^37,86,87^ γPNA-based inhibitors provide new avenues for developing miR therapeutics for clinical translation. Indeed, one such inhibitor effectively targets miR-210 for cancer therapy.^88^ Previous studies employing metabolic and cytokine analyses support the *in vivo* biocompatibility of γPNAs.^51,57,88^ However, little progress has been made in targeting miRs for cardiometabolic disorders. Our observation shows that neither the short (24 h)- nor long (6 week)-term exposure of γP-122-I is toxic (Figures 5 and 6) supports the biocompatibility of γPNAs.

γPNAs are highly resistant to cleavage by nucleases and proteases, which are highly substrate specific, and thus, they are not degraded inside the cell and form a highly stable duplex.^89^ Prior studies established that, on average, adding each γ modified nucleobase in the PNA increases the thermal binding of a PNA–RNA duplex by 5 °C.^40^ The thermal denaturation temperature of duplexes was significantly higher when γP-122-I, versus C-122-I, bound to miR-122, supporting the expectation that the affinity of γP-122-I for miR-122 is stronger (Figure 1D). miR-122 is found in the serum with argonaute2, the main component of the RNA-induced silencing complex, and can be internalized by neuropilin-1-expressing endothelial cells.^90−92^ It promotes endothelial cell apoptosis and is a risk factor for endothelial dysfunction.^93−95^ A recent report shows that the inhibition of miR-122 prevents atherosclerosis in ApoE^-/-^ mice.^34^ Here, we observed that the systemic administration of γP-122-I rescued endothelial dysfunction and improved glycemic control. The experimental and clinical studies show a positive association between serum miR-122 and hyperglycemia.^20,33,67,96^ Recently, we found that miR-122 regulates the expression of proinflammatory miR-204 in vascular endothelial cells.^33^ miR-204 is highly expressed in vascular endothelial cells,^33,64^ pancreatic β-cells,^97,98^ and cardiomyocytes.^101^ The inhibition or genetic deletion of miR-204 improves endothelial function and glycemic control despite obesity in the genetically diabetic *db*/*db* mice.^33,99^ Castaño et al. reported that the systemic administration of serum exosomes isolated from obese mice overexpressed miR-122 and promoted obesity and glucose intolerance in the lean mice by regulating PPAR-α in the epididymal white adipose tissue.^67^ Further, the HFD-fed mice overexpress PPAR-α in liver,^65^ and those lacking PPAR-α are protected from HFD-induced hyperglycemia.^66^ We also noted that γP-122-I reversed HFD-induced increase in PPAR-α levels in the liver. Therefore, the effects of miR-122 inhibition on miR-204 and PPAR-α are the potential mechanism through which γP-122-I improves the endothelial function and glycemic control in HFD-fed mice. As superior glycaemic control can itself improve endothelial function,^100^ it is possible that the observed γP-122-I-associated improvement in endothelial function is a consequence of a combination of miR-122 inhibition in the aorta and improved glycemic control. The high glucose condition decreases eNOS activation.^69,70^ Our results show that miR-122 inhibition partially rescues a high-glucose-induced increase in the eNOS expression and a decrease in the eNOS activation in HUVECs (Figure S4), supporting that improvement in endothelial function by miR-122 inhibition at least in part contributes to the improved endothelial function.

In conclusion, these results show that the γPNA-based miR-122 inhibitor γP-122-I improves vascular endothelial function and glycemic control without showing any evidence of toxicity *in vivo*. The overarching inference of this study is that the γPNA technology can be employed to generate next-generation miR inhibitors that are efficient and safer.

## Experimental Section

### General Experiments

Institutional Animal Care and Use Committee of the University of Iowa approved animal experiments and were performed according to National Institutes of Health (NIH) guidelines. All mice were maintained in a pathogen-free environment at the University of Iowa. C57BL/6 mice aged 8–16 weeks were used for the experiments. Eight-week-old mice were fed an HFD (TD.88137, Envigo, IN; containing 21.2% (w/w) fat, 48.5% (w/w) carbohydrate, 17.3% (w/w) protein, and 0.2% (w/w) cholesterol) for 8 weeks, and 2 weeks after this diet was initiated, they were injected with either γP-122-I or γP-SC (5 mg kg^–1^ day^–1^, intraperitoneal route) for 6 weeks. Age-matched ND-fed mice serve as controls. All compounds that were *in vivo* tested (γP-122-I and γP-SC) were >95% pure by HPLC (Figure S1). The area under the curve for single peaks from RP-HPLC traces for γPNA oligomers and the absence of any failure sequences ensure that γPNAs are >95% pure.

### Design and Synthesis of γP-SC and γP-122-I

*tert*-Butyloxycarbonyl (BOC)-protected diethylene glycol γ monomers were used for γP-122-I were procured from ASM Research Chemicals (Hannover, Germany). The monomers were vacuum-dried prior to the start of solid-phase synthesis. Approximately 100 mg of lysine-loaded resin was soaked in dichloromethane (DCM) for 5 h in a reaction vessel. DCM was drained, and the resin was deprotected using a mixture of trifluoroacetic acid and *m*-cresol for 5 min. This deprotection step was repeated twice, followed by washing the resin with DCM and *N*,*N*-dimethylformamide (DMF). The monomer was dissolved in a coupling solution comprising 0.2 M *N*-methyl pyrrolidone (NMP), 0.52 M di-isopropylethylamine (DIEA), and 0.39 M *o*-benzotriazole-*N*,*N*,*N*′,*N*′-tetramethyl-uroniumhexafluoro-phosphate (HBTU). The coupling solution was added to the reaction vessel and rocked for 2 h. The resin was capped using a capping solution (mixture of NMP, pyridine, and acetic anhydride) and then washed with DCM (8×). The entire process was repeated until the last monomer was added. 5-Carboxytetramethylrhodamine (TAMRA) was conjugated to the N terminus of γP-122 I. γPNA was cleaved from the resin using a cleavage cocktail (thioanisole, *m*-cresol, TMFSA, TFA (1:1:2:6)), and the vessel was rocked for 1.5 h. The γPNA was then collected and precipitated using diethyl ether, centrifuged at 3500 rpm for 5 min, washed with diethyl ether twice, and vacuum-dried. γPNA were purified by HPLC and its absorbance was measured by Nanodrop (Thermofisher Scientific, MA). The extinction coefficients of the individual monomers used to calculate the PNA concentration were 6600 M^–1^ cm^–1^ (C), 13 700 M^–1^ cm^–1^ (A), 8600 M^–1^ cm^–1^ (T), and 11 700 M^–1^ cm^–1^ (G).

### Vascular Reactivity

Vascular reactivity was determined as previously described.^33^ Briefly, aortic rings (thoracic aorta,1.5–2.0 mm wide) were placed in an ice-cold oxygenated (95% O_2_/5% CO_2_) Krebs–Ringer bicarbonate solution. The rings were placed in oxygenated organ bath filled with the KB solution. The organ baths were maintained at 37 °C. Each ring was suspended in a myograph system (DMT Instruments, FL). The extent of endothelium-dependent vasorelaxation was determined by generating dose–response curves to acetylcholine (Ach, 10^–9^–10^–5^ M) on aortic rings that had been precontracted by administering isotonic or isometric phenylephrine (PE, 10^–6^ M). Endothelium-independent vasorelaxation was determined by creating dose–response curves to SNP on aortic rings that had been precontracted with PE (10^–6^ M). Vasorelaxation (elicited by acetylcholine and SNP) was represented as a percentage of relaxation, calculated by dividing the inhibition ratio by the precontracted tension. Aortic rings that did not react to KCl or demonstrated autorelaxation were eliminated.

### Cell Culture

Human umbilical vein endothelial cells (Cat. No. CC-2519) were procured from Lonza (Mapleton, IL) and cultured in EGM-2 (Walkersville, MD) supplemented with growth factor. Cells were treated with high glucose (25 mM) for 24 h to simulate hyperglycemic conditions. As an osmolarity control, mannitol (25 mM) was utilized.

### qPCR

RNA was isolated using Trizol. miRs and RNAs were converted to cDNA using the qScript microRNA cDNA Synthesis Kit (Quanta bio). qPCR for miR-122 and TNF-α was performed using the SYBR Green RT-qPCR Kit, and 18S rRNA was used as an internal control. Serum miR levels were quantified using a constant amount of serum (200 μL).

### Gel-Shift Assays

miR-122 was incubated with PNAs (150 mM KCl, 2 mM MgCl_2_, 10 mM Na_3_PO_4_; pH 7.4) at 37 °C in a thermal cycler (T100, Bio-Rad, Hercules, CA) for 18 h. Samples were then separated on a 10% nondenaturing polyacrylamide gel using 1× tris/borate/EDTA buffer (1× TBE). After electrophoresis, the gels were stained with SYBR Gold (Invitrogen) in 1× TBE buffer for 2 min and imaged using a Gel-Doc EZ imager (Bio-Rad, Hercules, CA).

### Histological Processing and Immunostaining

The sections of formalin-fixed paraffin-embedded tissues were heated (95 °C) for 20 min in citrate buffer, followed by incubation with primary antibodies. For immunofluorescence experiments, anti-HIF-α (Thermofisher-MA1–516) and anti-CD45 (BD Pharmigen-610297) antibodies were used. Images were captured using Zeiss LSM 510. The histological sections were 5 μm thick and were stained using hematoxylin and eosin, and the images were captured using the Olympus microscope (BX-61).

### Measurement of Body Weight and Blood Glucose Levels and Performance of Intraperitoneal GTT

The body weight and blood glucose levels in ND, HFD-saline, HFD-γP-SC, and HFD-γP-122-I mice were measured at regular intervals (every 2 weeks). The mice fasted for 6 h, and their fasting blood glucose levels were measured. For IPGTT, the mice were injected intraperitoneally with glucose solution (2 g/kg) 6 h after fasting, and glucose levels were measured at 30, 60, 90, and 120 min time points after glucose injection. The white adipose tissue (epididymal, WAT) and brown adipose tissue (interscapular, BAT) were collected and weighed. Adiposity was calculated as the combined weight of WAT and BAT per 100 g of body weight.

### Statistical Analysis

GraphPad Prism was used for the statistical analysis (version 9.1). To establish the significance of the difference between the two groups, the *t*-test was performed. For multiple comparisons, ANOVA was utilized, and Tukey’s test was used for posthoc analysis. Nonlinear regression was used to assess the significance of the difference between the two vascular relaxation curves. The results were presented as mean ± SEM and were considered significant if *p* values were <0.05.

## Supporting Information Available

Purity and identification of γP-SC, γP-122-I, and TAMRA γP-122-I by HPLC and MALDI (Figure S1), time-dependent changes in TAMRA- γP-122-I levels (Figure S2), effect of γP-122-I on body weight, adiposity, and PPAR-α expression (Figure S3), and effect of miR-122 inhibition on eNOS and ERK1/2 expression and activation under hyperglycemic conditions (Figure S4) (PDF)

## Abbreviations Used

CBC: complete blood count
HCT: hematocrit
HFD: High-fat diet
HGB: hemoglobin
HPLC: high-performance liquid chromatography
MALDI: Matrix-assisted laser desorption/ionization
MCHC: mean corpuscular hemoglobin concentration
MCV: mean corpuscular volume
γPNA: γ-peptide nucleic acid
RBC: red blood cells
SNP: sodium nitroprusside
WBC: white blood cells
