# Supplementary material for: γ Peptide Nucleic Acid-Based miR-122 Inhibition Rescues Vascular Endothelial Dysfunction in Mice Fed a High-Fat Diet
Source: J Med Chem. 2022 Feb 8;65(4):3332–42. doi: 10.1021/acs.jmedchem.1c01831 (PMC8883473; doi:10.1021/acs.jmedchem.1c01831)
Supplement: Supplementary file 1 — jm1c01831_si_001.pdf [file jm1c01831_si_001.pdf]

Supporting Information

γ peptide nucleic acid-based miR-122 inhibition rescues vascular endothelial dysfunction in mice fed a high-fat diet

Ravinder Reddy Gaddam,[1#] Karishma Dhuri,[2#] Young-Rae Kim,[1] Julia S. Jacobs,[1] Vikas Kumar,[2] Qiuxia Li,[1] Kaikobad Irani,[1] Raman Bahal,[2*] Ajit Vikram[1*]

[1]Department of Internal Medicine, Carver College of Medicine University of Iowa, Iowa City, IA-52242, USA

[2]Department of Pharmaceutical Sciences, University of Connecticut, Storrs, CT-06269, USA

#These authors contributed equally to this work

*Corresponding authors

Raman Bahal: Assistant Professor of Pharmaceutics, Department of Pharmaceutical Sciences, University of Connecticut, Email: raman.bahal@uconn.edu

Ajit Vikram: Assistant Professor of Internal Medicine, Division of Cardiovascular Medicine, The University of Iowa, Carver College of Medicine, Phone: 319-335-2153, Email: ajit-vikram@uiowa.edu

Figure S1. **Purity and identification of γPNAs.** (A) High-performance liquid chromatography (HPLC) of γP-SC, γP-122-I, and TAMRA γP-122-I. (B) Matrix-Assisted Laser Desorption/Ionization (MALDI) spectrometry of γP-SC, γP-122-I, and TAMRA γP-122-I.

Figure S2. Time-dependent changes in levels of TAMRA- γP-122-I. Inhibitor levels in (A) serum and (B) urine, as determined by measurement of fluorescence (561 nm). Data are shown as mean, and the error bar represents s.e.m.

Figure S3. Effects of γP-122-I on body weight, adiposity, and hepatic PPAR-α. (A) Effect of HFD feeding and γP-122-I on the body-weight of mice. n = 5. (B) Effects of HFD feeding and γP-122-I treatment on adiposity. n = 5. (C) Effect of HFD feeding and γP-122-I on the PPAR-α expression in the liver. n = 4-6. WAT: white adipose tissue; BAT: brown adipose tissue; BW: body weight. ns $p > 0.05$, *$p < 0.05$ vs. indicated group. Data are shown as mean, and the error bar represents s.e.m.

Figure S4. Effects of C-122-I on the eNOS and ERK1/2 expression and activation in HUVECs. (A) Representative immunoblots showing the levels of p-eNOS, eNOS, p-ERK1/2, ERK1/2, and GAPDH in HUVECs under hyperglycemic conditions (25mmol/L, 24 hours) treated with either C-122-I or scrambled control (SC) (20nM). M-SC; cells treated with mannitol and SC, G-SC; cells treated with glucose and SC, G-C-122-I; cells treated with glucose and C-122-I. (B) Quantification of eNOS, p-eNOS, ERK1/2, and p-ERK1/2 in the HUVECs. n = 3-5. ns $p > 0.05$, *$p < 0.05$ vs. indicated group. Data are shown as mean, and the error bar represents s.e.m.

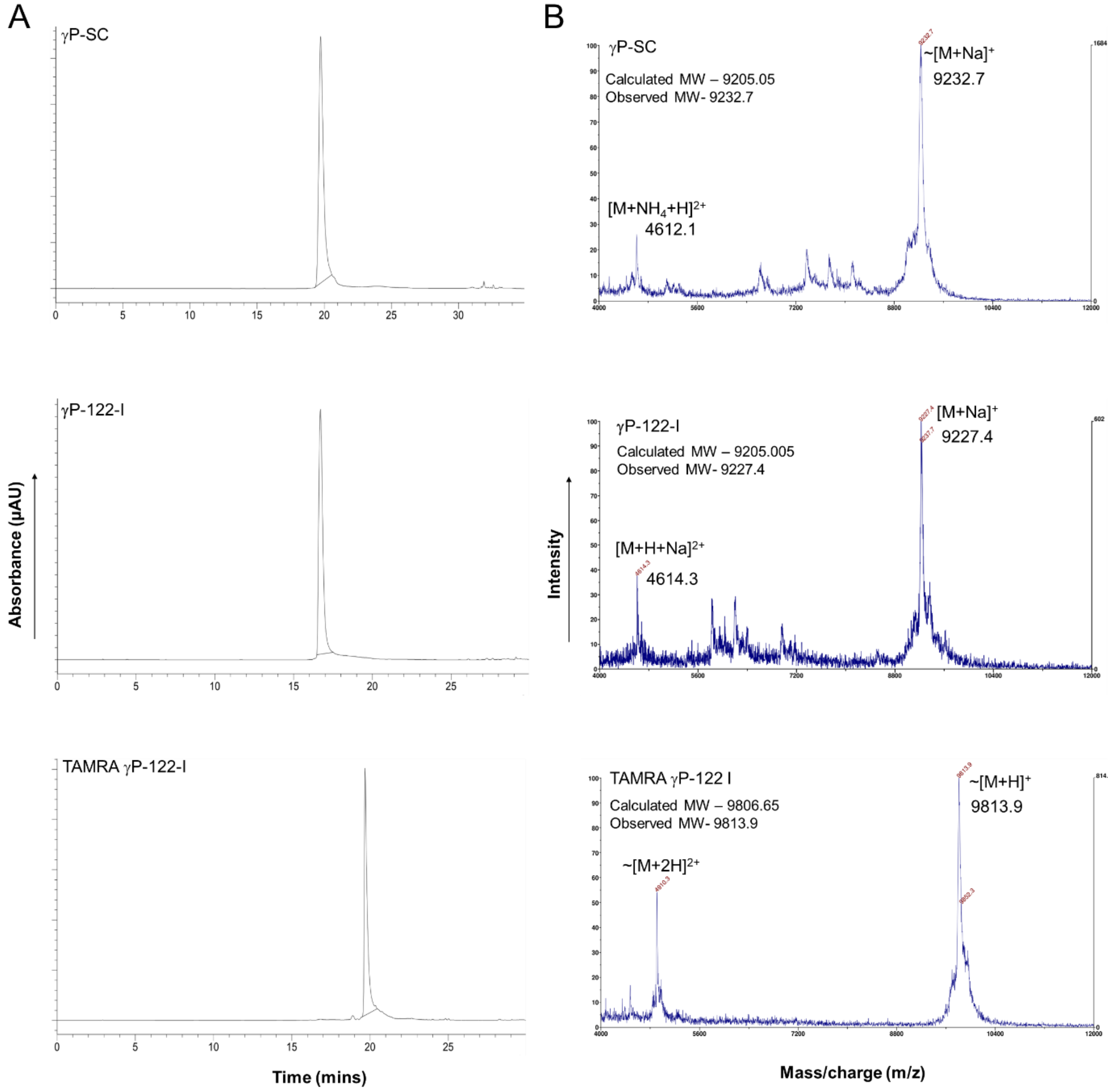

Figure S1. **Purity and identification of γPNAs.** (A) High-performance liquid chromatography (HPLC) of γP-SC, γP-122-I, and TAMRA γP-122-I. (B) Matrix-Assisted Laser Desorption/Ionization (MALDI) spectrometry of γP-SC, γP-122-I, and TAMRA γP-122-I.

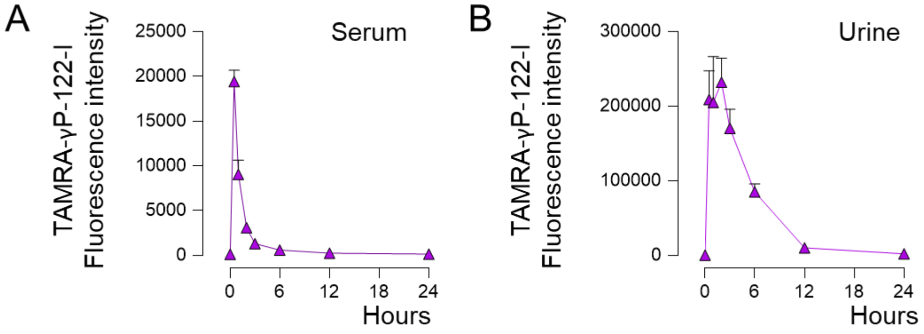

Figure S2. Time-dependent changes in levels of TAMRA- γP-122-I. Inhibitor levels in (A) serum and (B) urine, as determined by measurement of fluorescence (561 nm). Data are shown as mean, and the error bar represents s.e.m.

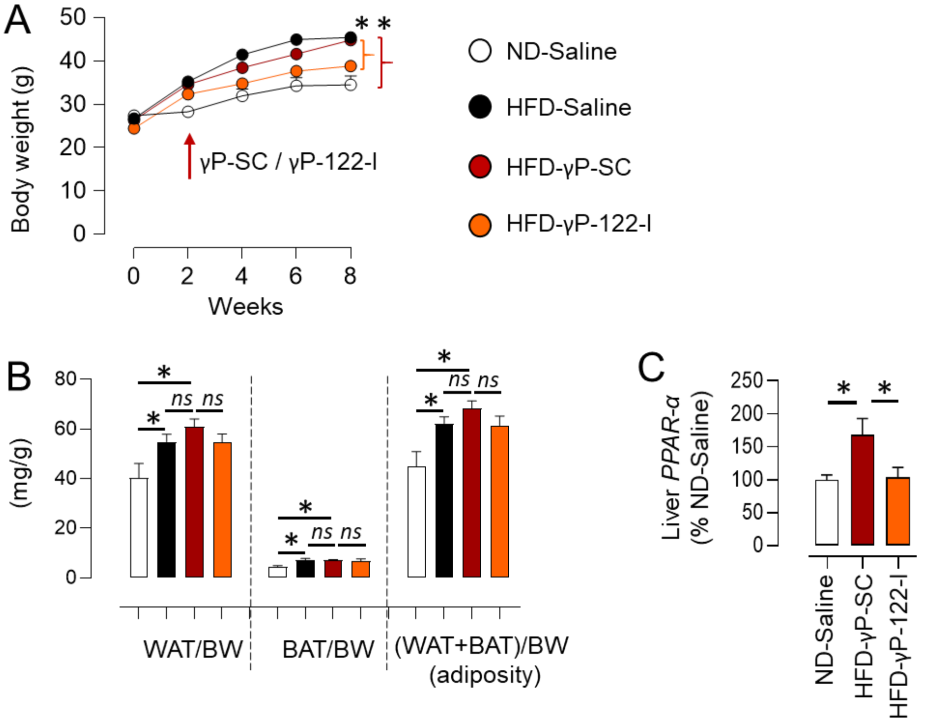

Figure S3. Effects of γP-122-I on body weight, adiposity, and hepatic PPAR-α. (A) Effect of HFD feeding and γP-122-I on the body-weight of mice. n = 5. (B) Effects of HFD feeding and γP-122-I treatment on adiposity. n = 5. (C) Effect of HFD feeding and γP-122-I on the PPAR-α expression in the liver. n = 4-6. WAT: white adipose tissue; BAT: brown adipose tissue; BW: body weight. ns p > 0.05, *p < 0.05 vs. indicated group. Data are shown as mean, and the error bar represents s.e.m.

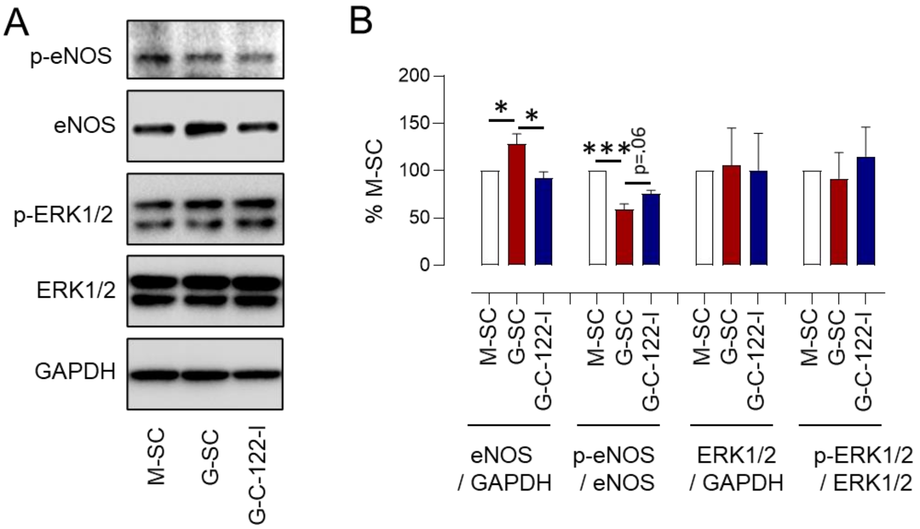

Figure S4. Effects of C-122-I on the eNOS and ERK1/2 expression and activation in HUVECs. (A) Representative immunoblots showing the levels of p-eNOS, eNOS, p-ERK1/2, ERK1/2, and GAPDH in HUVECs under hyperglycemic conditions (25mmol/L, 24 hours) treated with either C-122-I or scrambled control (SC) (20nM). M-SC; cells treated with mannitol and SC, G-SC; cells treated with glucose and SC, G-C-122-I; cells treated with glucose and C-122-I. (B) Quantification of eNOS, p-eNOS, ERK1/2, and p-ERK1/2 in the HUVECs. n = 4. ns p > 0.05, *p < 0.05, *p < 0.001 vs. indicated group. Data are shown as mean, and the error bar represents s.e.m.
